# Pain Medication Prescribing Patterns in Augmentation Mammoplasty

**DOI:** 10.1177/22925503211034828

**Published:** 2021-10-21

**Authors:** Jessica Winter, Braden Cruise, Blair R. Peters, Avi Islur

**Affiliations:** 1 Section of Plastic Surgery, Department of Surgery, Rady College of Medicine, University of Manitoba, Winnipeg, Manitoba, Canada; 2 Undergraduate Medical Education, Rady College of Medicine, University of Manitoba, Winnipeg, Manitoba, Canada; 3 First Gland Cosmetic Clinic, Manitoba, Winnipeg, Manitoba, Canada

**Keywords:** breast, breast augmentation, harm reduction, opioid analgesia, prescribing safety

## Abstract

**Background:**

The rate of opioid prescribing after low-risk surgical procedures has increased over the past decade, and surgeons are responsible for prescribing approximately one-third of all opioid medications. There is additional supporting evidence that patients only consume about half of the opioids prescribed to them after outpatient plastic surgery. Currently, there is no literature to provide surgeons with reference ranges for how much opioid medication will adequately provide analgesia for patients after undergoing bilateral breast augmentation (BBA) surgery.

**Objective:**

To quantify the amount of opioid medication required to adequately control pain for patients after undergoing BBA and use these data to provide recommendations on opioid prescribing practices.

**Methods:**

Cross-sectional prospective data were obtained through a take-home medication and pain tracking questionnaire for 56 patients after they underwent either subpectoral or subglandular BBA. Patients documented their pain scores on a 0 to 10 analogue scale and documented the type and amount of pain medication they took for a 7-day period.

**Results:**

Our study demonstrated that patients in the subglandular BBA group required an average of either 25 ± 1.2 Tylenol #3 or 19.3 ± 2.3 Tramacet tablets, and the subpectoral group required 27.7 ± 1.7 Tylenol #3 or 25.6 ± 0.9 Tramacet tablets over a 7-day period. There was no statistically significant difference between the 2 surgical groups.

**Conclusion:**

We propose a reference range of medication required on average for patients undergoing BBA to obtain adequate pain control in the initial postoperative period that falls within the most recent Canadian guidelines for safe opioid prescribing practices.

## Abstract (fr)

**Contexte:**

La fréquence de prescription des opioïdes après des procédures chirurgicales à faible risque a augmenté au cours de la dernière décennie et les chirurgiens sont responsables de la prescription d’environ un tiers de tous les médicaments opioïdes. Des données probantes supplémentaires indiquent que les patients ne consomment qu’environ la moitié des opioïdes qui leur ont été prescrits après une chirurgie plastique en chirurgie de jour. Aucune publication ne procure, à ce jour, des plages de référence aux chirurgiens pour leur indiquer combien d’opioïdes fournira aux patients une analgésie adéquate après avoir subi une chirurgie bilatérale d’augmentation mammaire.

**Objectif:**

Quantifier les médicaments opioïdes requis pour contrôler efficacement la douleur chez les patients ayant subi une chirurgie bilatérale d’augmentation mammaire et utiliser ces données pour fournir des recommandations sur les pratiques de prescription des opioïdes.

**Méthodes:**

Des données prospectives transversales ont été obtenues au moyen d’un questionnaire à remplir à domicile de suivi des médicaments et de la douleur auprès de 56 patientes venant de subir une chirurgie d’augmentation mammaire sous-pectorale ou sous-glandulaire. Les patientes ont documenté leurs scores de douleur sur une échelle analogique de 0 à 10, ainsi que le type et la quantité de médicament antidouleurs pris pendant une période de 7 jours.

**Résultats:**

Notre étude a démontré que les patientes du groupe augmentation mammaire sous glandulaire a nécessité une moyenne de 25 ± 1,2 comprimés de Tylénol #3 ou 19,3 ± 2,3 comprimés de Tramacet; les patientes du groupe sous-pectoral ont nécessité 27,7 ± 1,7 comprimés de Tylénol #3 ou 25,6 ± 0,9 comprimés de Tramacet comprimés sur une période de sept jours. Il n’y a pas eu de différence statistiquement significative entre les deux groupes chirurgicaux.

**Conclusion:**

Nous proposons une plage de référence pour les médicaments nécessaires en moyenne pour les patientes subissant une chirurgie d’augmentation mammaire pour contrôler correctement la douleur au cours de la période postopératoire initiale; cette plage de référence correspond aux plus récentes lignes directrices canadiennes sur les pratiques de prescription sécuritaire des opioïdes.

## Introduction

Recently, there has been greater scrutiny and growth in concern toward opioid prescribing practices citing increased use and abuse of these medications. This has resulted in a growing push toward greater regulation on opioid prescriptions.^1^ For over a decade, Canada and the United States have seen a steady increase in the number of prescriptions and the use of opioid analgesics.^2^ Although opioids are considered in the World Health Organization pain ladder for the treatment of postoperative pain management, they are not the first line of therapy.^3^ The difficulty of providing adequate analgesics for the management of pain while balancing the risk of abuse presents a significant challenge to plastic surgeons, with detection of abuse often remaining difficult.^4^ Surgeons are responsible for nearly 37% of all opioid prescriptions, and the rate of opioid prescribing after low-risk surgical procedures has increased in the last decade.^5^ Numerous studies have concluded that even short-term exposure to opioids in the postoperative period puts patients at risk of developing an opioid use disorder.^5^ Standard amounts of postoperative opioid prescriptions for outpatient surgery have not been explored in the literature.^1^ Additionally, no evidence exists for standard opioid prescribing patterns within the bilateral breast augmentation (BBA) population. With a lack of available guidance to surgeons on prescription practices, it is no surprise that recent literature has found that patients only consume half of the opioids prescribed to them after outpatient plastic surgery. This indicates an opportunity to reduce opioid prescribing within the plastic surgery community.^6^ Improved understanding of opioid consumption after elective surgery may lead to improved prescribing, lower costs, and less leftover medication available for potential misuse.^7^

## Purpose

This study aims to objectively obtain data to quantify the amount of opioid medication required to adequately control pain for patients undergoing BBA for both subpectoral and subglandular procedures.

## Methods

Research ethics board approval was obtained to approach patients who met inclusion criteria to participate in the research study. Informed consent was obtained from all participants. All procedures performed in studies involving human participants were in accordance with the ethical standards of the institutional and/or national research committee and with the 1964 Helsinki declaration and its later amendments or comparable ethical standards. The authors of this article have no conflicts of interest to disclose.

Study inclusion criteria were as follows: All patients undergoing elective primary BBA, received a post-operative outpatient prescription for pain control (nonsteroidal anti-inflammatory drugs or narcotics), speak, read and write English, and older than 18 years of age.

Patients were excluded from participating in the study if they were undergoing revision procedures, undergoing additional operations to the breast elsewhere, had a history of opioid addiction/abuse, were currently taking high doses of opioid medication or opioid antagonists, or were currently diagnosed with chronic pain conditions.

Patients who met inclusion criteria were recruited over a 1-year period, January 2019 to January 2020.

Cross-sectional prospective data, using purposeful sampling, were obtained through a take-home patient pain tracking questionnaire. Fifty-six patients were instructed to fill out a numeric visual analogue scale (0-10) to rate their overall pain on that day and track the types and amount of pain medication they took for 7 days. Simple statistics were performed.

All patients underwent an inframammary fold crease incision, and dissection was carried out with electrocautery. A dual plane (I or II) was performed for all submuscular implant placements, and a standard subglandular (not subfascial) plane was created for those undergoing subglandular augmentation. Intraoperatively, 10 cm^3^ of 0.25% Marcaine was injected into the incision site and along the lateral pectoral muscle border (for intercostal block) in each breast. All patients were placed into a postoperative compression bra prior to discharge and instructed to take opioid medications only as necessary. All patients receiving Tylenol #3 in the submuscular group were provided with an additional prescription for cyclobenzaprine (10 mg po twice daily prn) postoperatively to reduce muscular cramping. Those patients receiving Tramacet could not take cyclobenzaprine due to potential drug interaction. All surgery was performed by a single surgeon.

From an anaesthetic point, patients received dexamethasone 8 mg intravenously (IV) once intraoperatively as an antiemetic. No muscle relaxant was given. Toradol 30 mg IV was given in a single dose postoperatively if there were no issues with bleeding. Exceptions to this would be if the patient had a history of sensitivity or allergy to the above medication.

Patients were encouraged to start nonopioid analgesia (NOA; Advil, Motrin, Tylenol, or Alieve) 48 hours postoperatively and to only take prescribed (Tylenol #3 or Tramacet) postoperative medication as required. Prescribed opioid analgesia was selected based off patient preference with no history of allergy, and prescriptions were written as formulary standard. Patients were encouraged to ween opioid medication as early as possible. The active ingredient of Tylenol #3 is codeine, 15 mg per tablet. The active ingredient in Tramacet is tramadol, 37.5 mg per tablet. Both codeine and tramadol are morphine derivatives. Patients were encouraged to start mobilization on postoperative day 0, with no restriction in arm movements. Many returned to work immediately. For the first 2 weeks postoperatively, the patients were instructed to refrain from activity that would increase their blood pressure or heart rate significantly.

## Results

The average age for the subpectoral group was 34.4 years (range: 20-62 years of age). The average age for the subglandular group was 38.9 years (range: 24-68 years of age). The average body mass index (BMI) for the subpectoral group was 22.5 range: 16.9-38.3 years of age). Average BMI for the subglandular group was 23.9 (range: 19.1-42.1 years of age). In the subpectoral group, there were 11 nulliparous patients out of 27 patients. In the subglandular group, there were 8 nulliparous patients out of 29 patients. Averaging the number of pills taken by patients over a 7-day period, comparing nulliparous women to primiparous/multiparous women was not significantly different in either group.

Within the subglandular BBA group, the average patient required 25 ± 1.2 Tylenol #3 tablets per person (56.3 morphine equivalents), 19.3 ± 2.3 Tramacet tablets (72.4 morphine equivalents), and/or 26.5 ± 0.9 NOA tablets per person over the first 7 days postoperatively. The subpectoral BBA group average tablets per patient were 27.7 ± 1.7 Tylenol #3’s (62.3 morphine equivalents), or 25.6 ± 0.69 Tramacets (96 morphine equivalents), and/or 24 ± 0.3 NOA tablets.

Although the morphine equivalent requirements were found to be higher in the submuscular group, there was no statistically significant difference between the 2 surgical groups in the amount of Tylenol #3 or Tramacet tablets that were taken (*P* =.66 and *P* = .29, respectively; Table 1).

**Table 1.** Average Amount of Opioid and Nonopioid Pain Medication Taken by Study Participants.

	Tylenol #3	Tramacet	NOA
Subglandular (N = 29)	25 ± 1.2 tablets 56.3 morphine equivalents	19.3 ± 2.3 tablets 72.3 morphine equivalents	26.5 ± 0.9 NSAIDs
Subpectoral (N = 27)	27.7 ± 1.7 tablets 62.3 morphine equivalents	25.6 ± 0.9 tablets 96.0 morphine equivalents	24.0 ± 0.3 NSAIDs

Abbreviations: NOA, nonopioid analgesia; NSAIDs, nonsteroidal anti-inflammatory drugs.

**Figure 1.**
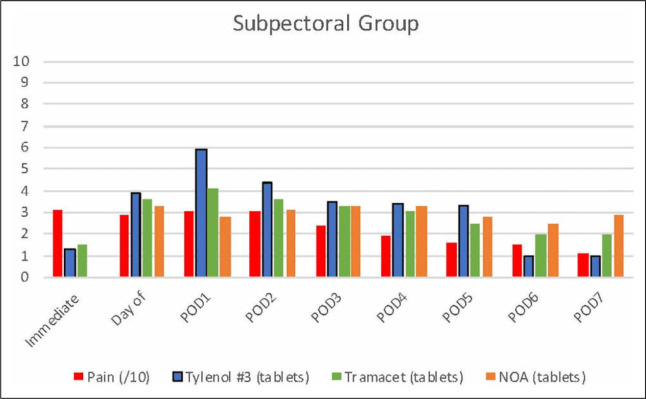
Bar graph representing average pain and medication use postoperatively following subglandular augmentation mammoplasty.

**Figure 2.**
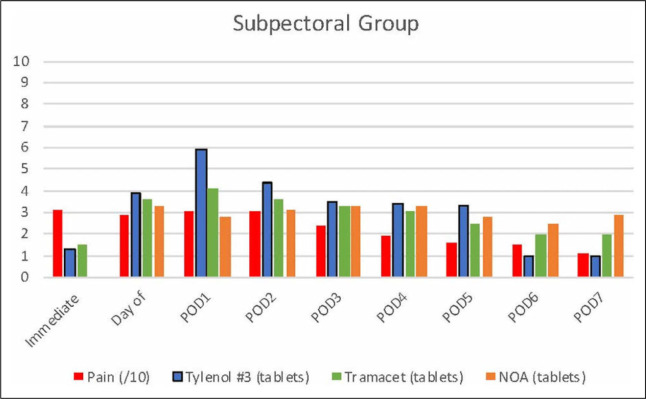
Bar graph representing average pain and medication use postoperatively following subpectoral augmentation mammoplasty.

Both groups had adequate and comparable self-reported pain control rated 3.1/10 on average throughout the 7-day postoperative period (Figures 1 and 2). Only 28% and 40% of participants in the subglandular and subpectoral groups, respectively, accessed the use of NOA during the postoperative period. Nonopioid analgesia included celecoxib 200 mg tablets, acetaminophen 650 mg tablets, ibuprofen 400 mg tablets, and naproxen 250 mg tablets.

## Discussion

Canada has the second-highest rate of prescription opioid use worldwide, behind the United States.^7^ Death-related opioid overdoses have increased 8-fold over the past 2 decades, resulting in significant public health concern.^8^ The most current Canadian guidelines for opioids thoroughly cover the use and safe prescribing practices for the use of opioid analgesics in chronic noncancer pain in both the general and special populations such as those with substance abuse disorders or mental illness.^9^ Key points from the recommended guidelines include optimizing nonopioid therapy and avoiding opioids in patients with substance abuse altogether whenever possible. For patients beginning opioid therapy, the Canadian guidelines recommend restricting to less than 90 mg morphine equivalents daily (MED) and suggest restricting the maximum prescribed dose to less than 50 mg MED. As well they stress the importance of tapering and recognizing those who are having difficulty in tapering should be promptly referred a multidisciplinary program.^9^ The overprescribing of opioid analgesia, particularly within the postoperative period, has played a significant role in the current opioid epidemic. It is estimated that 71% of opioid pills went unused, and 92% of patients had pills left over after surgery.^10^

Of importance, author’s wish to highlight that when given alone, opioids are not the most effective method of treating postoperative pain. Early use of NOA as multimodal therapy and early weaning of opioid analgesia have been well-established in the literature as first-line harm reduction and methods to reduce the risk of dependence.^7,11^

Our study found that no difference between the subpectoral and subglandular groups. Anecdotally, this opposes current views. Patients who fell within the subpectoral group generally received a muscle relaxant (cyclobenzaprine 10 mg po twice daily prn) postoperatively versus those in the subglandular group that did not. This intervention may explain our findings and requires further investigation. Although we did not find a statistically significant difference between the 2 groups over the 7-day postoperative period, we did find that the subpectoral group took longer to discontinue the use of opioid analgesia.

Several other areas within plastic surgery are being investigated to establish safe prescribing patterns, particularly for routine and standardized procedures. In hand surgery, Rodgers et al^2^ demonstrated that a prescription of 30 opioid pills for outpatient surgery appeared to be excessive and recommended a prescription of 15 tablets with one refill.^7^ Merola et al identified a gross amount of the overprescribing of opioid therapy following bilateral reduction mammoplasty, in their 24 594 patient retrospective cohort study they concluded that those who received over 60 MED were more likely to refill prescriptions and develop dependence in the perioperative period. This was statistically significant when compared to those who received 15 to 59 MED in the perioperative period.^12^ To mitigate the risk of dependency, some groups have advocated for using an Opioid Risk assessment tool upon initial evaluation.^11^

A limitation of our study as with any self-administered questionnaires is self-reporting bias or type 3 error. Patients may not have been entirely transparent and could have either under-reported or exaggerated their medication usage and pain scores for fear of judgement or simply forgot to report their numbers one day leading to false reporting. However, it was to the surgeon’s best knowledge to select for reliable patients for the survey, as well from a surgical candidacy point. A recent study by Rose et al similarly aims to quantify opioid consumption patterns for outpatient plastic surgery for those who underwent breast augmentation consumed a range of 62 to 94 MED.^6^ These findings are similar to our findings for the subpectoral group, they did not specify surgical technique.

Another limitation of the study design is the inability to accurately assess the daily dosages of the nonopioid analgesics. The self-reported data included the quantity of nonopioid medications taken but it did not specify the dosage of the tablets or capsules. In addition, 3 questionnaires had to be discarded as they were not properly filled out.

Additionally, due to underpowered numbers, conclusions could not be drawn in regard to BMI and whether this was variable in greater postoperative pain, only 6 patients fell outside of the normal BMI classification (18.5-24.9).

Further research into data collection for standardized surgical procedures may lead to more informed and safer opioid prescribing habits for surgeons. Greater numbers would be required to standardize pill quantity. Additionally, our study showed a relatively low number of patients using multimodal drug therapy in both the subglandular and subpectoral groups, 28% and 40%, respectively. Perhaps more education to patients and surgeons around the use of multimodal therapy would reduce opioid consumption in surgical patients.

## Conclusion

We propose a reference range of medication required on average for patients undergoing BBA to obtain adequate pain control in the initial postoperative period; this works out to be approximately 24 to 30 tablets of Tylenol #3 and 17 to 26 tablets of Tramacet. Patients should be counselled and encouraged to engage in multimodal therapy and weaning of opioid analgesia as soon as possible. The authors found that opioid requirements following BBA were less than 50 morphine equivalents per day, in keeping with the most recent Canadian guidelines.^9^
